# Employing the structural equation modeling in assessing urban farming innovation adoption: examining urban society’s acceptance of the theory of planned behavior and technology acceptance model frameworks

**DOI:** 10.3389/fsoc.2026.1808040

**Published:** 2026-05-15

**Authors:** E. T. Yuniarsih, Muslim Salam, Muhammad Hatta Jamil, A. Nixia Tenriawaru

**Affiliations:** 1 Doctoral Program of Agricultural Sciences, Graduate School of Hasanuddin University, Makassar, Indonesia; 2 Research Center for Behavioral and Circular Economics, Research Organization for Governance, Economy, and Community Welfare, National Research and Innovation Agency, South Jakarta, Indonesia; 3 Laboratory of Agricultural Development, Department of Socio-economics of Agriculture, Faculty of Agriculture, Hasanuddin University, Makassar, Indonesia; 4 Laboratory of Agribusiness, Department of Socio-economics of Agriculture, Faculty of Agriculture, Hasanuddin University, Makassar, Indonesia

**Keywords:** adoption technology, structural equation modeling, technology acceptance model, theory of planned behavior, urban farming

## Abstract

**Introduction:**

This study examined the factors influencing the adoption of urban farming technologies in urban communities in Makassar City, Indonesia. It integrated the Theory of Planned Behavior (TPB) and the Technology Acceptance Model (TAM) to provide a comprehensive understanding of how psychological, social, and technological factors shape adoption intentions.

**Methods:**

Primary data were collected from 346 respondents across 15 subdistricts using a questionnaire survey. The data collection was supported by agricultural extension officers who served as field facilitators, having received prior guidance to ensure consistent data collection. The data were analyzed using Partial Least Squares Structural Equation Modeling (PLS-SEM).

**Results:**

The results revealed that knowledge, subjective norms, attitudes, perceived behavioral control, perceived usefulness, and perceived ease of use significantly influenced the intention to adopt urban farming. Several variables also played mediating roles, particularly perceived ease of use, which links socio-demographic characteristics and behavioral control to adoption intention. In addition, subjective norms are influenced by both ease of use and perceived control.

**Discussion:**

These findings highlight the importance of integrated strategies, including community-based education, strengthening social support systems, and improving access to user-friendly technologies. Such efforts are essential to enhancing public participation and promoting the sustainable adoption of urban farming practices.

## 1 Introduction

Rapid global urbanization has increased pressure on urban food systems and necessitates more sustainable food production strategies. In this context, urban farming is increasingly seen as a potential approach to improving local food security, shortening food distribution chains, and enhancing urban environmental sustainability (Pennisi et al., 2022). Previous studies have shown that urban farming practices can improve access to fresh food and strengthen household resilience. However, the level of adoption among urban communities remains uneven. Several studies emphasize that land constraints, access to technology, and policy support are key factors influencing the development of urban agriculture (Erälinna and Szymoniuk, 2021). More recent studies also indicate that individual social and psychological factors, such as attitudes toward innovation, social norms, and perceptions of technology’s benefits, play an important role in shaping people’s decisions to adopt urban farming practices (Yuniarsih et al., 2024).

Although these studies have provided valuable insights into the development of urban farming, most prior research has primarily focused on technical, environmental, or policy aspects of urban agriculture development (De Bon et al., 2010; Ilieva et al., 2022; Pennisi et al., 2022). Research that specifically examines the behavioral mechanisms influencing urban communities’ intentions to adopt urban farming practices, particularly through an integrated theoretical approach combining psychological factors and technology acceptance, remains limited (Shao et al., 2022; Siddiqui et al., 2024). Furthermore, the relationship between individual behavioral factors and perceptions of technology in the context of urban agricultural innovation adoption has not yet been comprehensively examined within a unified analytical framework (Yu and Tao, 2009; Sun et al., 2021).

To address this gap, this study adopts an integrated behavioral framework that combines the Theory of Planned Behavior (TPB) developed by Ajzen (1991) and the Technology Acceptance Model (TAM) developed by Davis (1989). The TPB explains that an individual’s behavioral intention is influenced by attitudes toward the behavior, subjective norms, and perceived behavioral control. Meanwhile, TAM explains technology acceptance through two main constructs: perceived usefulness and perceived ease of use, which reflect the extent to which a technology is considered beneficial and easy to use. The integration of TPB and TAM provides a more comprehensive analytical framework by incorporating psychological, social, and technological factors in explaining individuals’ intentions to adopt urban farming practices.

By integrating these two theoretical frameworks, this study examined how psychological factors and perceptions of technology jointly influence urban communities’ intentions to adopt urban farming practices. This study aims to contribute to the literature on urban farming adoption by developing an integrated behavioral model that combines psychological perspectives and technology acceptance to explain people’s intentions to adopt urban farming practices. Specifically, this study tested the hypothesis that attitudes toward urban farming, subjective norms, and perceived behavioral control positively influence individuals’ intentions to adopt urban farming practices. In addition, perceived usefulness and perceived ease of use are also hypothesized to positively influence individuals’ intentions to adopt urban farming practices.

## 2 Conceptual framework of TAM and TPB

An individual’s background and level of knowledge influence how they respond to technological innovations, including urban farming practices. In behavioral literature, the Theory of Planned Behavior (TPB) explains that an individual’s intention to engage in a behavior is influenced by attitude toward the behavior, subjective norms, and perceived behavioral control (Ajzen, 1991). This framework has been widely used to explain individual decisions regarding the adoption of innovative practices, including in the agricultural sector and urban food systems. Recent studies indicate that psychological and social factors play a significant role in shaping community decisions to engage in urban agriculture and sustainable food innovations (Elbeheiry and Balog, 2022; Pennisi et al., 2022). In an urban context, such decisions are influenced not only by individual preferences but also by social norms and community interactions within the urban social environment.

On the other hand, the Technology Acceptance Model (TAM) emphasizes that technology acceptance is influenced by individuals’ perceptions of usefulness and ease of use (Davis, 1989). These constructs explain how individuals evaluate the practical benefits and ease of use of a technology before deciding to adopt it. Technologies such as hydroponics, vertical farming, and other technology-based food production systems require individuals to evaluate their benefits and ease of implementation in daily life. Studies indicate that perceived benefits and ease of use are critical factors in driving the adoption of agricultural innovations, including within urban farming (Erälinna and Szymoniuk, 2021; Yuniarsih et al., 2024).

Although both models are widely used in innovation adoption studies, using a single model in isolation often fails to fully explain decision-making dynamics in complex social contexts such as urban environments. TPB emphasizes psychological and social factors, while TAM focuses on individuals’ perceptions of technological characteristics. Therefore, integrating these two models provides a more comprehensive analytical framework for understanding how psychological factors, social norms, and perceptions of technology jointly influence individuals’ intention to adopt urban farming practices.

**Figure 1:**
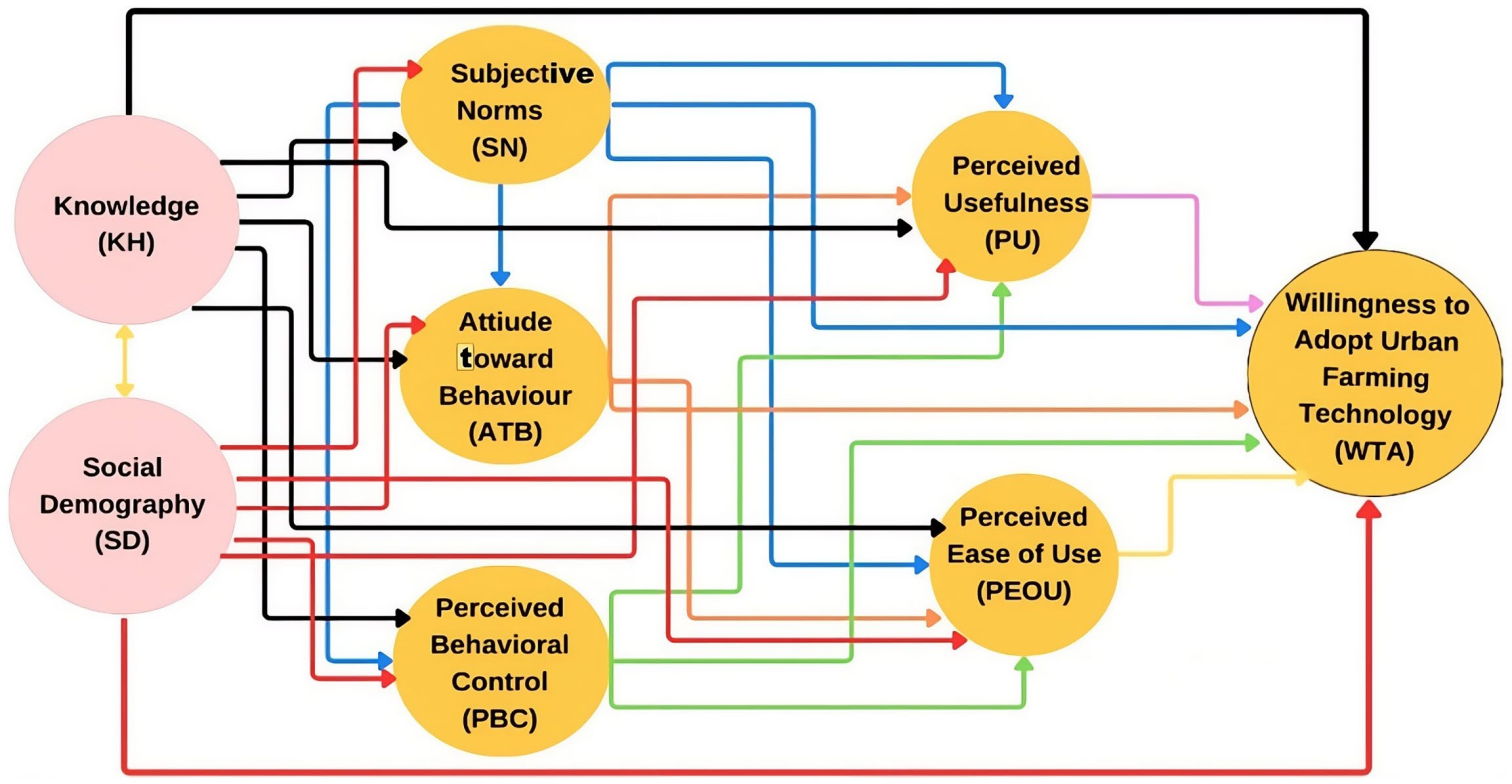
Conceptual framework of the research integrating the Theory of Planned Behavior (TPB) and the Technology Acceptance Model (TAM).

In addition to the core constructs in TPB and TAM, this study also includes knowledge (KH) and socio-demographic characteristics (SD) as exogenous variables. Knowledge represents an individual’s level of understanding of urban farming concepts and practices, which can influence attitudes and perceptions toward the technology’s benefits. Meanwhile, socio-demographic characteristics such as age, educational level, and socioeconomic status reflect structural factors that can influence an individual’s access to information, resources, and social networks supporting urban agriculture practices. Thus, KH and SD are positioned as antecedent factors influencing the constructs within TPB and TAM before ultimately shaping the public’s behavioral intention to adopt urban farming innovations. The conceptual framework of this study, which illustrates the relationships among variables in this integrative model within the context of urban farming in Makassar City, is presented in Figure 1.

## 3 Research method

### 3.1 Research location and sample size

**Figure 2:**
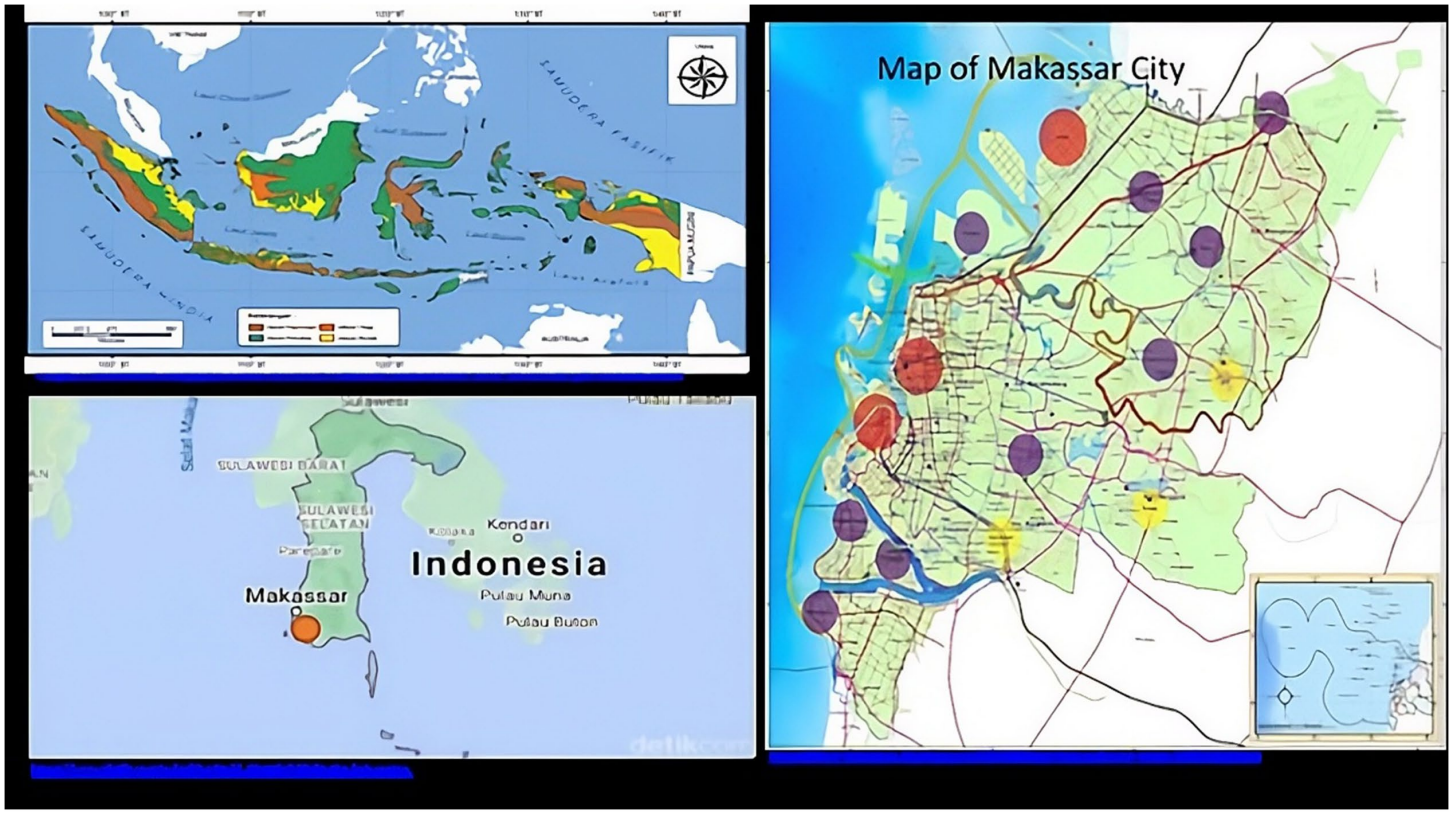
Map of the research location in Makassar City, Indonesia. Source: Adapted and compiled by the authors from multiple publicly available geographic and regional data sources, including: https://ipsgampang.blogspot.com/2014/08/bentuk-muka-bumi-dan-aktivitas-penduduk.html; https://www.detik.com/sulsel/berita/d-6344547/daftar-24-kabupaten-; https://timur-nusantara.blogspot.com/2012/08/rtrw-kota-makassar-tahun-2010-2030.html.

This study was conducted in Makassar City, South Sulawesi Province, Indonesia, one of the major economic and urban centers in Eastern Indonesia. The study location was selected because Makassar has experienced rapid population growth, exhibits significant social, economic, and cultural diversity, and demonstrates strong local government support for urban farming through initiatives such as the Lorong Garden and Taman Lorong programs. Data collection was carried out from April to July (Yue et al., 2023). As shown in Figure 2, the map illustrates the administrative boundaries of Makassar City, including the main road network and the distribution of study locations. This study covers 15 subdistricts in Makassar City, which are marked with bullet symbols on the map. These markers represent the distribution of data collection sites across various parts of the city, reflecting the diverse characteristics of Makassar’s urban areas in the context of urban farming implementation.

The conceptual framework of this study integrates variables from the Technology Acceptance Model (TAM) and the Theory of Planned Behavior (TPB), which have been widely used in research on technology adoption and individual behavior (Ajzen, 1991; Davis, 1989; Qin et al., 2011; Sun et al., 2021; Siddiqui et al., 2024). Both models have been widely recognized as effective in explaining factors influencing individuals’ intentions and behaviors toward technology adoption across various research contexts. Variables derived from TAM include Perceived Usefulness (PU) and Perceived Economic Benefit (PEB), while variables from the TPB model include Attitude toward Behavior (ATB), Subjective Norm (SN), and Perceived Behavioral Control (PBC). Additionally, this study incorporates sociodemographic (SD) characteristics and knowledge variables to complement the analysis of social factors and public understanding that may influence the adoption of urban farming practices. Based on these constructs, the research indicators were operationalized into a series of questionnaire items tailored to the context of urban farming practices in urban communities.

All research instruments were drafted in Indonesian to ensure that each question was clearly understood by respondents, in accordance with the local social and cultural context. Using a survey questionnaire, primary data were gathered from 346 respondents across 15 subdistricts. The questionnaire was distributed with the assistance of agricultural extension workers serving as field assistants. Prior to data collection, the extension workers received a structured briefing (coaching) regarding the research objectives, questionnaire content, and data collection procedures to ensure consistency in implementation. The extension workers distributed the questionnaire to the respondents across the 15 subdistricts of Makassar City. Respondents were recruited randomly from the urban communities within these areas, particularly individuals who were relevant and engaged in urban farming. This approach enabled broader respondents, improved response rates, and ensured that the captures diverse socio-economic characteristics of urban communities in the study area. In total, seven variables were measured in this study: subjective norm, attitude, perceived behavioral control, perceived usefulness, perceived economic benefit, sociodemographics, and knowledge. The sample size was determined using Yamane’s formula (Osahon and Kingsley, 2016) (Equation 1).


$$n=(\frac{N}{1+Ne^{2}})$$ (1)

Where: *n* = sample size, *N* = total population, *e* = sampling error (1% or 5%).

The calculation yielded a sample of 346 respondents from the city’s total population of 1,427,620 (Statistics of Makassar Municipality, 2022), with a 5% margin of error at a 95% confidence level.

### 3.2 Data analysis

#### 3.2.1 Structural equation modeling analysis (SEM): empirical model specification

**Figure 3:**
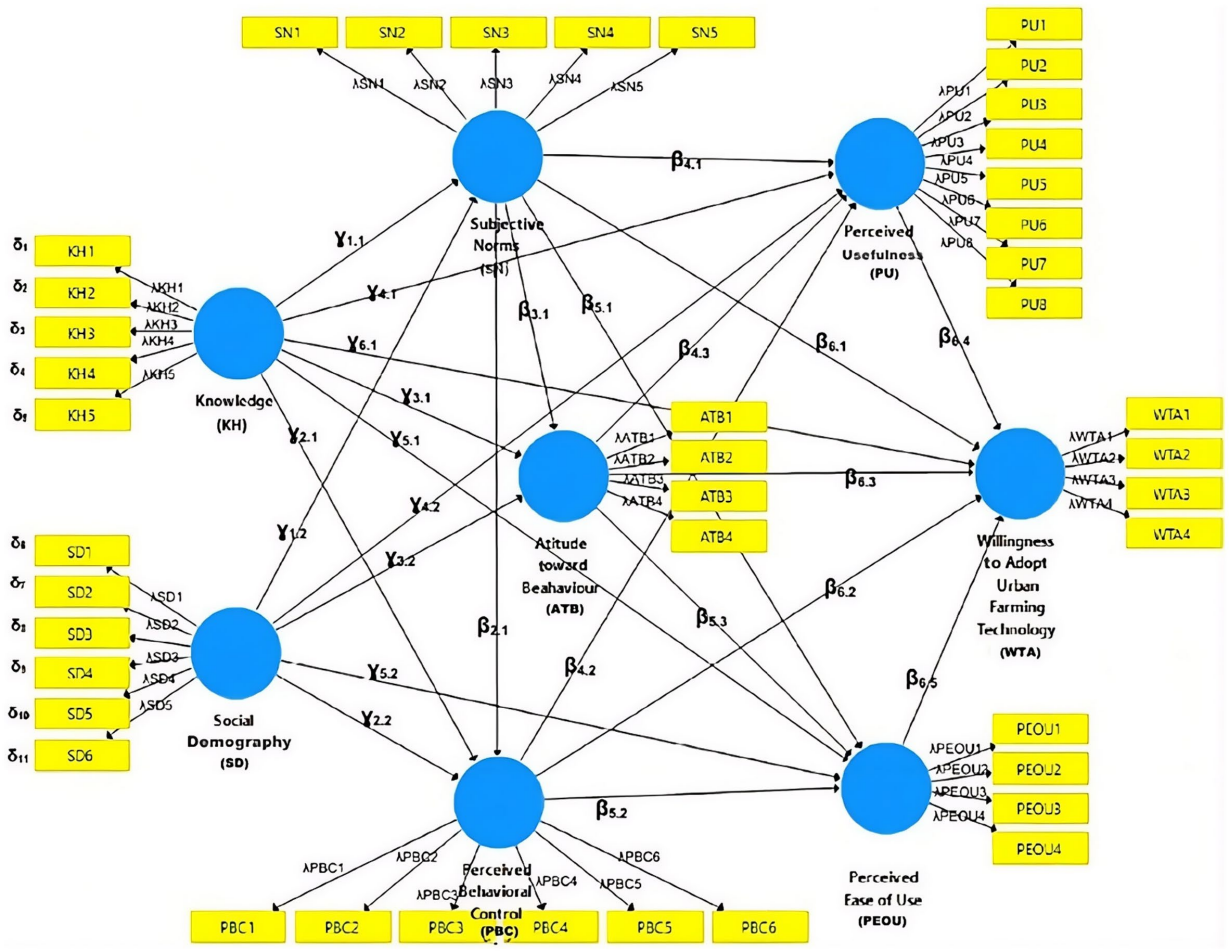
Path diagram of the theoretical framework based on the TAM and TPB models.

This study applied Partial Least Squares Structural Equation Modeling (PLS-SEM), a variance-based approach suitable for exploratory analysis, to estimate the model and assess its predictive capability (Kock, 2024). The procedure included tests of measurement validity, reliability, and structural relationships (Sudirman Bisri et al., 2024). The model and its indicators are presented in Figure 3 and Table 1.

**Table 1:** Description of latent and indicator variables, and their measurement units.

Variable latent	Indicator variables	Symbols	References	Measurement unit^*^
Exogenous latent variables				
Knowledge (KH)	Knowledge of the types of technologies in urban farming (UF)	KH1	Kaginalkar et al. (2023)	1–5-point scale
	UF technologies suitable for the residential environment	KH2	Jin et al. (2021)	1–5-point scale
	Knowledge that UF contributes to food availability and security	KH3	Talema and Nigusie (2023)	1–5-point scale
	Knowledge of resource use efficiency in UF	KH4		1–5-point scale
	Knowledge that UF practices help maintain environmental sustainability	KH5	Puppim de Oliveira et al. (2022)	1–5-point scale
Social demography (SD)	Age	SD1	Mashi et al. (2022)	CD: 1 = <25; 2 = 26–35; 3 = 35–45; 4 = 46–55; 5 = >56
	Education	SD2		CD: 1 = Primary School; 2 = Middle School; 3 = High School; 4 = Undergraduate Degree; 5 = Graduate/Doctoral Degree.
	Experience	SD3		Likert scale (1–5 points)
	Occupation	SD4		CD: 1 = Public Sector Worker, 2 = Private Sector Employee/Academic, 3 = Business Owner/Farmer/Consultant, 4 = Casual Laborer/Contract Worker, 5 = Homemaker/Retired
	Income	SD5	Abdoellah et al. (2023)	CD: 1 = <IDR 1,000,000 2 = IDR 1,000,000–2,999,999 3 = IDR 3,000,000–4,999,999 4 = IDR 5,000,000–9,999,999 5 = >IDR 10,000,000
	Number of family members	SD6	Rezadoost and Allahyari (2014) and Mashi et al. (2022)	CD: 1 = 1; 2 = 2–4; 3 = 3–7; 4 = 8–10; 5 = >10.
Endogenous latent variables of TPB				
Subjective norm (SN)	Inspiration gained from UF practices	SN1	Dutta and Chandrasekharan (2019)	1–5-point scale
	Social norms	SN2	Khan et al. (2023)	1–5-point scale
	Social support	SN3		1–5-point scale
	Government support	SN4	Gurbuz and Ozkan (2021)	1–5-point scale
	Opinions from community leaders	SN5	Glover (2004)	1–5-point scale
Perceived behavioral control (PBC)	Resource accessibility	PBC1		1–5-point scale
	Ability to perform behavior	PBC2	Shao et al. (2022)	1–5-point scale
	Control over external factors	PBC3	Kang et al. (2015)	1–5-point scale
	Behavioral planning	PBC4	Kristensen et al. (2004)	1–5-point scale
	Social control	PBC5		1–5-point scale
	Financial capability	PBC6		1–5-point scale
Attitude toward behavior (ATB)	Belief that UF will provide benefits	ATB1	Yusoff et al. (2017)	1–5-point scale
	Assessment/evaluation of the benefits gained from UF practices	ATB2	Keraita and Drechsel (2015)	1–5-point scale
	Personal norms	ATB3	Goh and Ho (2023)	1–5-point scale
	Risks in UF	ATB4	Mitchell et al. (2021)	1–5-point scale
Endogenous latent variable of TAM				
Perceived usefulness (PU)	Physical health benefits	PU1	Helen and Gasparatos (2020)	1–5-point scale
	Food quality benefits	PU2	Addas et al. (2024)	1–5-point scale
	Environmental sustainability benefits	PU3	Ohe (2007) and Cristiano (2021)	1–5-point scale
	Psychological benefits for individuals	PU4	Świąder et al. (2023)	1–5-point scale
	Educational benefits	PU5	Mayfield et al. (2023)	1–5-point scale
	Food security benefits	PU6	Addas et al. (2024)	1–5-point scale
	Food independence benefits	PU7		1–5-point scale
	Social relationship benefits	PU8	Ilieva et al. (2022)	1–5-point scale
Perceived ease of use (PEOU)	Availability of resources and clear guidelines	PEOU1	Koutridi and Christopoulou (2023)	1–5-point scale
	Level of ease of use	PEOU2	Liu et al. (2023)	1–5-point scale
	Feedback/support from extension workers	PEOU3	Ishak et al. (2022)	1–5-point scale
	Time required for learning UF practices	PEOU4	Rizal et al. (2019)	1–5-point scale
Willingness to adopt urban farming technologi (WTA)	Readiness to start urban farming	WTA1	Yu et al. (2023)	1–5-point scale
	Commitment to engage in urban farming	WTA2	Giannoccaro et al. (2022)	1–5-point scale
	Confidence in the ability to achieve success in UF practices	WTA3		1–5-point scale
	Understanding of food and environmental sustainability	WTA4	Cristiano (2021)	1–5-point scale

^*^CD, Categorical data.

#### 3.2.2 Stage of the research process

**Figure 4:**
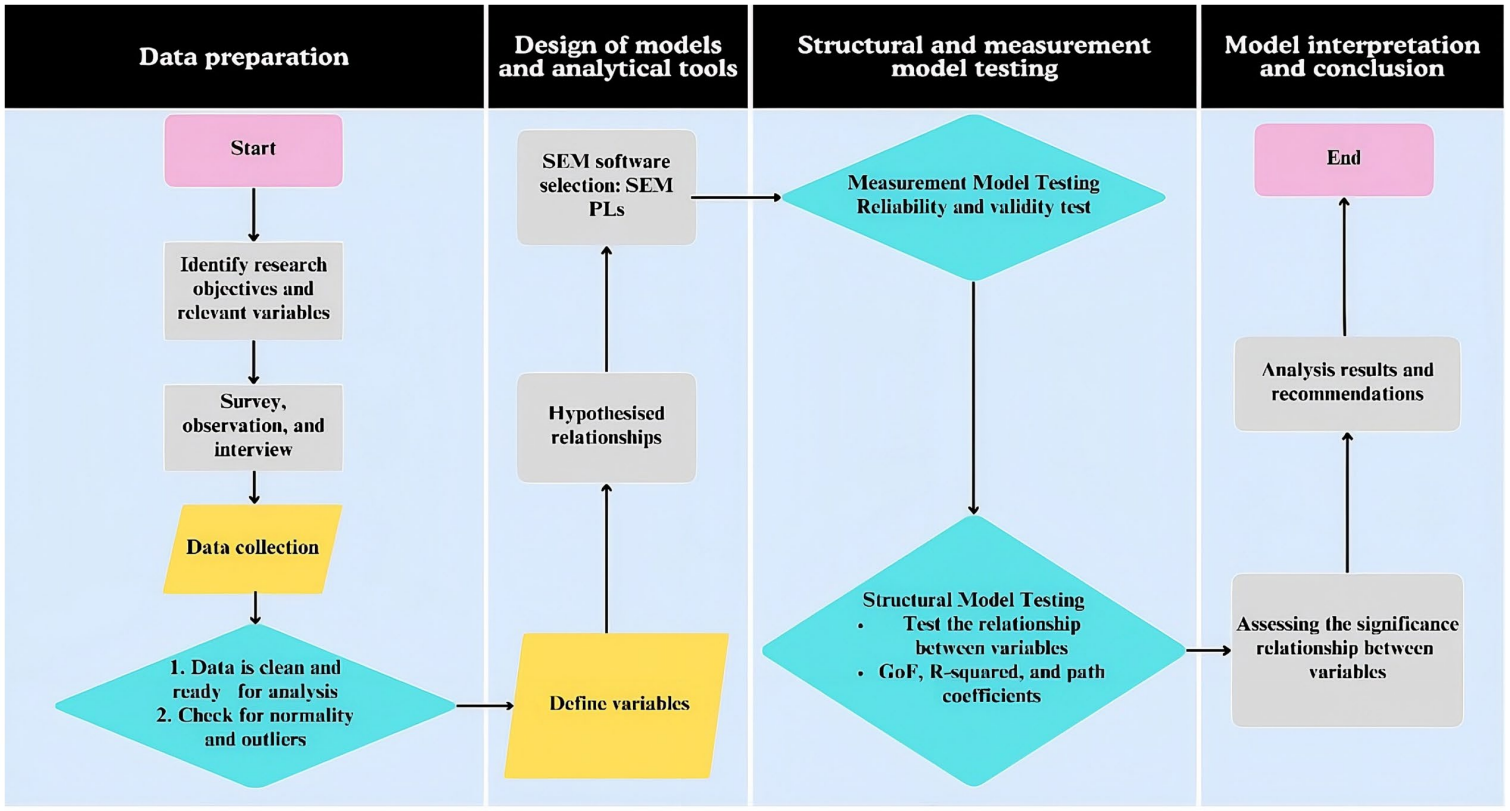
Flowchart of the research process.

The SEM analysis explored the relationships among variables, with the main analytical stages illustrated in Figure 4.

#### 3.2.3 Model design and analytical methods

Survey data were collected, cleaned to correct errors, screened for missing values and outliers, and adjusted as necessary to ensure data accuracy for SEM analysis. Latent constructs, observed indicators, and relationships among variables were determined based on a comprehensive literature review (Fu et al., 2024). Hypothesis testing was conducted to examine the effects of variables derived from the Technology Acceptance Model (TAM) and the Theory of Planned Behavior (TPB) variables on the adoption of urban farming using a significance threshold of *t* > 1.96 or *p* < 0.05. Definitions of constructs, measurement indicators, and their references are presented in Table 2. To ensure a comprehensive evaluation of the proposed model, the analysis was conducted through several main stages, including:

1. Outer model testing

**Table 2:** Hypotheses based on the literature review from previous studies.

Hypothesis	References	Analysis results
KH → WTA	Zhang and Luo (2023)	+
KH → PEOU	Wang and Chen (2021)	+
KH → SN	Liou et al. (2016)	+
KH → PU	Wang and Chen (2021)	+
KH → ATB	Otache et al. (2021)	+
KH → PBC	Siddiqui et al. (2024)	+
SD → SN	De Bon et al. (2010)	+
SD → ATB	Kim and Choi (2023)	+
SD → PBC	Cahyono (2023)	±
SD → PU	Sun et al. (2021)	+
SD → PEOU		+
SN → PU	Yu and Tao (2009)	+
SN → PBC	Prabowo et al. (2022)	±
SN → PEOU	Qin et al. (2011)	+
SN → ATB	Vieira et al. (2016)	+
SN → WTA	Baig et al. (2024)	+
ATB → PU	Ge et al. (2023)	+
ATB → WTA	King et al. (2023)	+
ATB → PEOU	Ge et al. (2023)	+
PU → WTA	Wagner et al. (2024)	+
PBC → PU	Amin et al. (2017)	+
PBC → PEOU		+
PBC → WTA	Li et al. (2022)	+
PEOU→WTA	Waked et al. (2024)	+

The symbol of “→” indicates an influence between variables. The abbreviations used are explained in Table 1. The symbol “±” indicates that the relationship between the variables can be either positive or negative.

The outer model was evaluated to assess construct validity and indicator reliability (Hair et al., 2013). The measurement model, along with a detailed description of latent variables and indicators, is presented in Figure 3 and Tables 1 and 3.

1. Inner model testing

**Table 3:** Structural and measurement equations in path diagram.

a. Outer model equation^*^			b. Inner model equation^*^
Exogenous latent variables	Endogenous latent variables		
Knowledge (KH)	Subjective norm (SN)	Perceived usefullnes (PU)	SN = ɣ_1.1_ KH + ɣ_1.2_ SD + ζ_1_
KH1 = λKH1 KH + *δ*1	SN1 = λSN1 SN + *ε*1	PU1 = λPU1 PU + ε16	PBC = ɣ_2.1_ KH + *β*_2.1_ SN + ζ_2_
KH2 = λKH2 KH + δ2	SN2 = λSN2 SN + ε2	PU2 = λPU2 PU + ε17	ATB = ɣ_3.1_ KH + ɣ_3.2_ SD + β_3.1_ SN + ζ_3_
KH3 = λKH3 KH + δ3	SN3 = λSN3 SN + ε3	PU3 = λPU3 PU + ε18	PU = ɣ_4.1_ KH + ɣ_4.2_ SD + β_4.1_ SN + β_4.2_ PBC + β_4.3_ ATB + ζ_4_
KH4 = λKH4 KH + δ4	SN4 = λSN4 SN + ε4	PU4 = λPU4 PU + ε19	PEOU = ɣ_5.1_ KH + ɣ_5.2_ SD + β_5.1_ SN + β_5.2_ PBC + β_5.3_ ATB + ζ_5_
KH5 = λKH5 KH + δ5	SN5 = λSN5 SN + ε5	PU5 = λPU5 PU + ε20	WTA = ɣ_6.1_ KH + β_6.1_ SN + β_6.2_ PBC + β_6.3_ ATB + β_6.4_ PU + β_6.5_ PEOU + ζ_6_
Social demography SD	Perceived behaviour control (PBC)	PU6 = λPU6 PU + ε21	
SD1 = λSD1 SD + δ6	PBC1 = λPBC1 PBC + ε6	PU7 = λPU7 PU + ε22	
SD2 = λSD2 SD + δ7	PBC2 = λPBC2 PBC + ε7	PU8 = λPU8 PU + ε23	
SD3 = λSD3 SD + δ8	PBC3 = λPBC3 PBC + ε8	Perceived ease of use (PEOU)	
SD4 = λSD4 SD + δ9	PBC4 = λPBC4 PBC + ε9	PEOU1 = λPEOU1 PEOU + ε24	
SD5 = λSD5 SD + δ10	PBC5 = λPBC5 PBC + ε10	PEOU2 = λPEOU2 PEOU + ε25	
SD6 = λSD6 SD + δ11	PBC6 = λPBC6 PBC + ε11	PEOU3 = λPEOU3 PEOU + ε26	
	Attitude toward behaviour (ATB)	PEOU4 = λPEOU4 PEOU + ε27	
	ATB1 = λATB1 ATB + ε12	Willingness to Adopt (WTA)	
	ATB2 = λATB2 ATB + ε13	WTA1 = λWTA1 WTA + ε28	
	ATB3 = λATB3 ATB + ε14	WTA2 = λWTA2 WTA + ε29	
	ATB4 = λATB4 ATB + ε15	WTA3 = λWTA3 WTA + ε30	

Descriptions: KH and SD are exogenous latent variables, while SN, PBC, ATB, PU, PEOU, and WTA are endogenous. δ and ε denote error terms for exogenous and endogenous variables, respectively. ɣ and β represent regression paths, and ξ indicates structural error. The symbol * in indicates statistical significance at the 5% level (*p* < 0.05).

The inner (structural) model was evaluated to examine relationships among latent constructs, with *R*^2^ used to measure predictive power (Jogiyanto, 2009). Structural relationships were evaluated using *R*^2^, *F*^2^, and *Q*^2^ values to assess construct influence and overall model performance.

1. Model interpretation and conclusion

In SEM, significance was determined based on path coefficients, *t*-values (>1.96), and *p*-values (<0.05). Model evaluation was conducted using *R*^2^, Chi-square, CFI, and RMSEA, while reliability and validity were confirmed through Cronbach’s Alpha and Composite Reliability, thereby ensuring the robustness of both the measurement and structural models.

## 4 Results and analysis

### 4.1 Outer model testing

**Figure 5:**
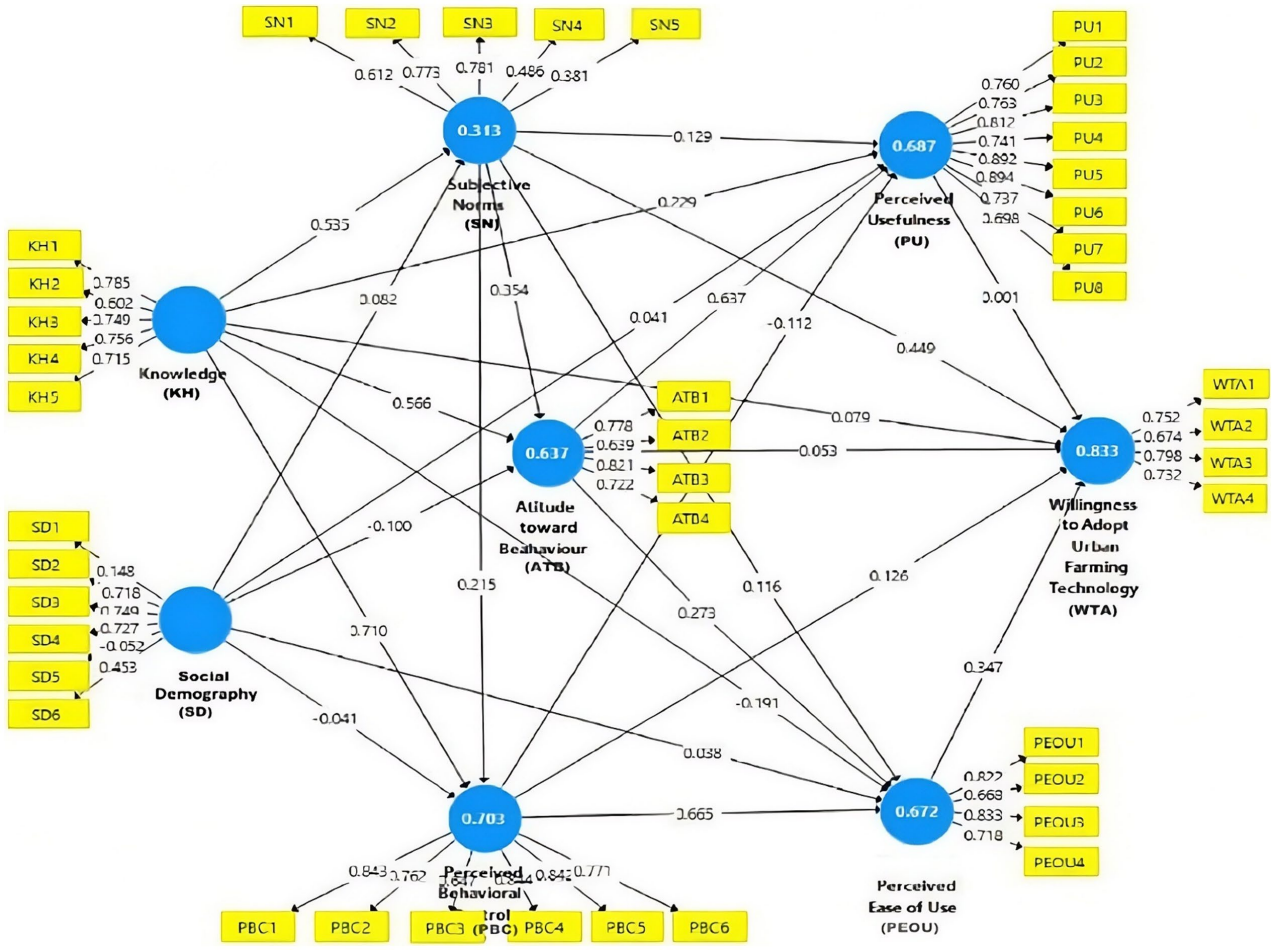
Initial outer loading results.

SmartPLS 3 was used to analyze the inner and outer models, examining the relationships among variables and the performance of indicators. The validity and reliability of the outer model were assessed using standard criteria (Hair et al., 2013) with loading values shown in Figure 5.

**Figure 6:**
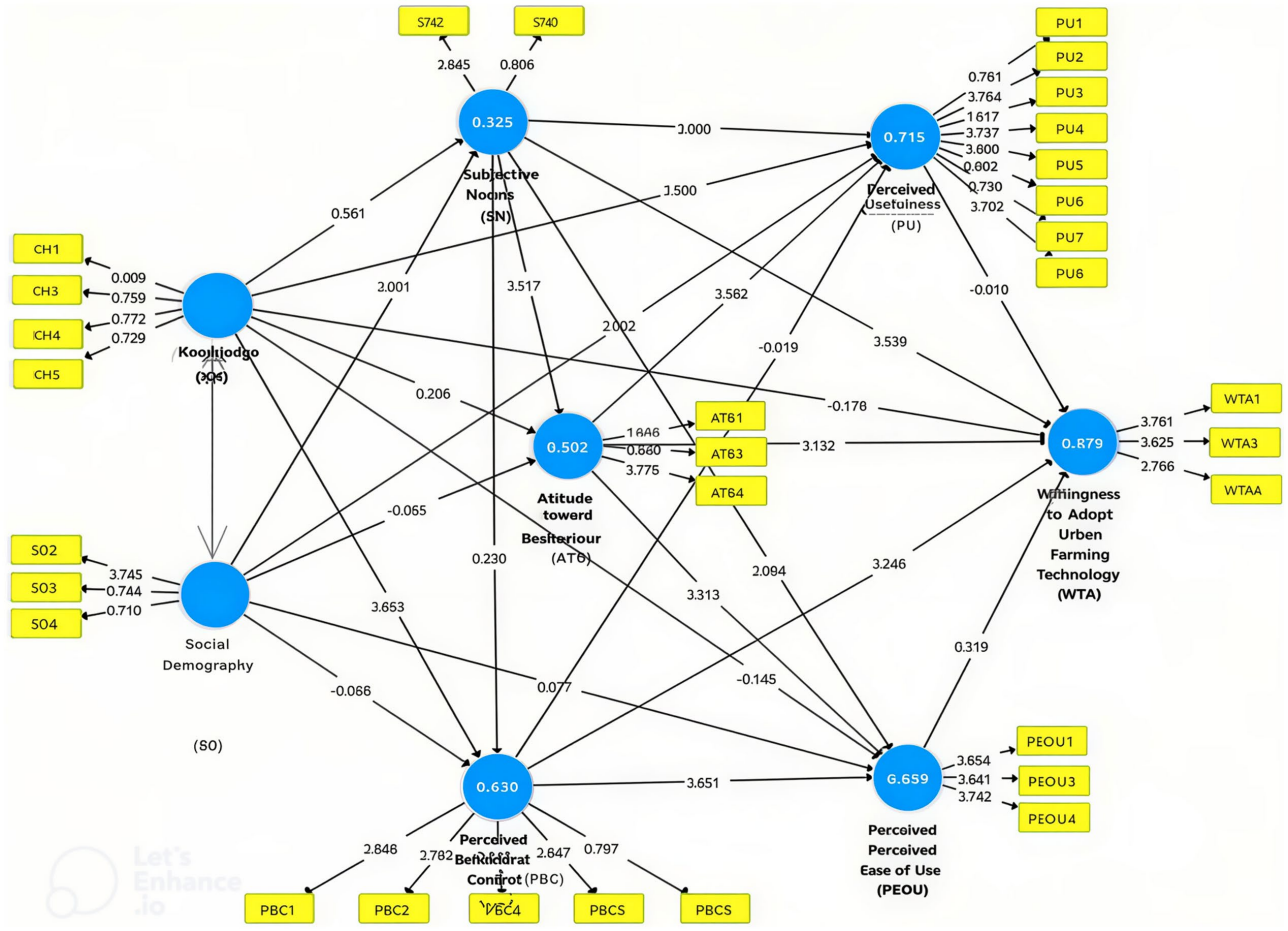
Outer loading analysis results after removing indicators KH2, SD1, SD5, SD6, SN1, SN4, SN5, PBC3, ATB2, PEOU2, and WTA2.

As shown in Figures 6, 11 indicators had loading factor values greater than 0.7, necessitating reanalysis after removing invalid indicators, as illustrated in Figure 5. Figure 5 indicates that 11 of the 42 indicators were eliminated because their outer loading values were below 0.7. Construct validity and reliability test revealed that the Knowledge variable (KH) demonstrated strong reliability, with an Average Variance Extracted (AVE) value of 0.589. The socio-demographic variable (SD) also exhibited acceptable validity, with an AVE of 0.537. Additionally, the Subjective Norm (SN) and Perceived Behavioral Control (PBC) variables had adequate AVE values of 0.682 and 0.684, respectively. Conversely, the Attitude toward Behavior (ATB) and Perceived Ease of Use (PEOU) variables required further refinement. Detailed results of validity and reliability tests for each indicator are presented in Table 4.

**Table 4:** The validation test results for each indicator.

Latent variables/research indicators	Outer loading	Average variance extracted (AVE)
Knowledge (KH)		0.589
Knowledge of the types of technologies in urban farming (UF) (KH1)	0.809	
Knowledge that UF contributes to food availability and security (KH3)	0.759	
Knowledge of resource use efficiency in UF (KH4)	0.772	
Knowledge that UF practices help maintain environmental sustainability (KH5)	0.729	
Social demography (SD)		0.537
Education (SD2)	0.745	
Experience (SD3)	0.744	
Occupation (SD4)	0.71	
Subjective norms (SN)		0.682
Social norms (SN2)	0.845	
Social support (SN3)	0.806	
Perceived behavioral control (PBC)		0.684
Resource accessibility (PBC1)	0.848	
Ability to perform behavior (PBC2)	0.792	
Behavioral planning (PBC4)	0.848	
Social control (PBC5)	0.847	
Financial capability (PBC6)	0.797	
Attitude toward behavior (ATB)		0.653
Belief that UF will provide benefits (ATB1)	0.808	
Personal norms (ATB3)	0.84	
Risks in UF (ATB4)	0.775	
Perceived usefulness (PU)		0.624
Physical health benefits (PU1)	0.761	
Food quality benefits (PU2)	0.764	
Environmental sustainability benefits (PU3)	0.812	
Psychological benefits for individuals (PU4)	0.737	
Educational benefits (PU5)	0.89	
Food security benefits (PU6)	0.892	
Food independence benefits (PU7)	0.739	
Social relationship benefits (PU8)	0.702	
Perceived ease of use (PEOU)		0.662
Availability of resources and clear guidelines (PEOU1)	0.854	
Feedback/support from extension workers (PEOU3)	0.841	
Time required for learning UF practices (PEOU4)	0.742	
Willingness to adopt UF (WTA)		0.617
Readiness to start urban farming (WTA1)	0.761	
Confidence in the ability to achieve success in UF practices (WTA3)	0.825	
Understanding of food and environmental sustainability (WTA4)	0.768	

The SEM-PLS results (Table 5) indicate acceptable to high reliability across constructs. The latent variables Knowledge (KH) and Perceived Usefulness (PU) demonstrated strong reliability (CR = 0.851 and 0.929). Socio-demography (SD) and Subjective Norms (SN) showed adequate reliability, although SN exhibited a slightly lower Cronbach’s Alpha (Schuberth, 2021). ATB, PEOU, Willingness to Adopt Urban Farming (WTA), and PBC also met reliability standards (CR = 0.849–0.910), confirming model validity and supporting subsequent analyses of urban farming adoption.

**Table 5:** Results of latent variable testing and *R*^2^.

Latent variables	Cronbach’s alpha	Rho_A	Composite reliability	*R* ^2^
KH	0.767	0.771	0.851	–
SD	0.643	0.609	0.777	–
SN	0.534	0.537	0.811	0.325
PBC	0.887	0.891	0.915	0.630
ATB	0.734	0.733	0.849	0.502
PU	0.912	0.914	0.929	0.715
PEOU	0.750	0.786	0.854	0.689
WTA	0.689	0.694	0.828	0.879

### 4.2 Inner model testing (*R*^2^)

The PLS-SEM results (Table 5) displayed varying *R*^2^ values across constructs. The latent variable Subjective Norms (SN) (*R*^2^ = 0.325) indicated moderate social influence (Khan et al., Yue et al., 2023), while Perceived Behavioral Control (PBC) (*R*^2^ = 0.630) reflected access to resources and financial capacity (Hurst et al., 2024). ATB (*R*^2^ = 0.502) was associated with beliefs and perceived risks (Kalula et al., 2024), whereas PU (*R*^2^ = 0.715) and PEOU (*R*^2^ = 0.689) were influenced by perceptions of health, sustainability, and resource availability (Yue et al., 2023). The Technology Adoption construct (TA) (*R*^2^ = 0.879) demonstrated strong predictive accuracy, driven by commitment and sustainability values.

### 4.3 Results of direct and indirect effect significance testing (*T*-test and *P*-test)

As shown in Table 6, Knowledge (KH) negatively affected Willingness to Adopt Urban Farming (WTA) (*β* = −0.178, *p* < 0.001), indicating that higher knowledge may foster skepticism. In contrast, ATB (*β* = 0.132, *p* = 0.003), PBC (*β* = 0.246, *p* < 0.001), and PEOU (*β* = 0.319, *p* < 0.001) positively influenced WTA, whereas PU was not significant (*β* = −0.019, *p* = 0.638). Bootstrapping results showed that KH indirectly affected WTA through PBC and PEOU (*β* = 0.136, *p* < 0.001), but not via PU. KH also directly increased PBC (*β* = 0.160, *p* < 0.001) while indirectly decreasing WTA via PBC (*β* = −0.046, *p* = 0.019).

**Table 6:** The direct and indirect effects of variables in the study.

Description	Original sample (O)	*T* statistics (|O/STDEV|)	*p* values
KH → WTA	−0.178	4.317	0.000^*^
SN → WTA	0.539	13.944	0.000^*^
ATB → WTA	0.132	2.990	0.003^*^
PBC → WTA	0.246	6.054	0.000^*^
PEOU → WTA	0.319	8.538	0.000^*^
PU → WTA	−0.019	0.471	0.638
KH → PBC → PU → WTA	0.000	0.149	0.882
KH → PBC → WTA	0.160	5.423	0.000^*^
KH → PEOU → WTA	−0.046	2.350	0.019^*^
KH → PU → WTA	−0.006	0.459	0.646
KH → SN → ATB → PEOU → WTA	0.029	4.445	0.000^*^
KH → SN → ATB → PU → WTA	−0.003	0.466	0.641
KH → SN → ATB → WTA	0.038	2.755	0.006^*^
KH → SN → PBC → PEOU → WTA	0.027	4.079	0.000^*^
KH → SN → PBC → PU → WTA	0.000	0.143	0.886
KH → SN → PBC → WTA	0.032	4.087	0.000^*^
KH → SN → PEOU → WTA	0.017	1.804	0.071
KH → SN → PU → WTA	−0.001	0.394	0.694
KH → SN → WTA	0.302	8.476	0.000^*^
PBC → PEOU → WTA	0.208	6.302	0.000^*^
PBC → PU → WTA	0.000	0.148	0.882
SD → ATB → PEOU →WTA	−0.006	1.429	0.153
SD → ATB → PU → WTA	0.001	0.404	0.686
SD → ATB → WTA	−0.007	1.224	0.221
SD → PBC → PEOU →WTA	−0.012	1.413	0.158
SD → PBC → PU → WTA	0.000	0.136	0.892
SD → PBC → WTA	−0.014	1.466	0.143
SD → PEOU → WTA	0.024	2.116	0.034^*^
SD → PU → WTA	0.000	0.262	0.793
SD → SN → ATB → PEOU → WTA	0.002	0.701	0.484
SD → SN → ATB → PU → WTA	0.000	0.252	0.801
SD → SN → ATB → WTA	0.002	0.649	0.517
SD → SN → PBC → PEOU → WTA	0.001	0.653	0.514
SD → SN → PBC → PU → WTA	0.000	0.080	0.937
SD → SN → PBC → WTA	0.002	0.665	0.506
SD → SN → PEOU → WTA	0.001	0.567	0.571
SD → SN → PU → WTA	0.000	0.215	0.830
SD → SN → WTA	0.017	0.700	0.484
SN → ATB → PEOU → WTA	0.052	5.245	0.000^*^
SN → ATB → PU → WTA	−0.006	0.470	0.638
SN → ATB → WTA	0.068	2.939	0.003^*^
SN → PBC → PEOU → WTA	0.048	4.188	0.000^*^
SN → PBC → PU → WTA	0.000	0.145	0.885
SN → PBC → WTA	0.057	4.411	0.000^*^
SN → PEOU → WTA	0.030	1.862	0.063
SN → PU → WTA	−0.002	0.395	0.693

The symbol of “→” indicates an influence between variables. The symbol * in indicates statistical significance at the 5% level (*p* < 0.05).

**Figure 7:**
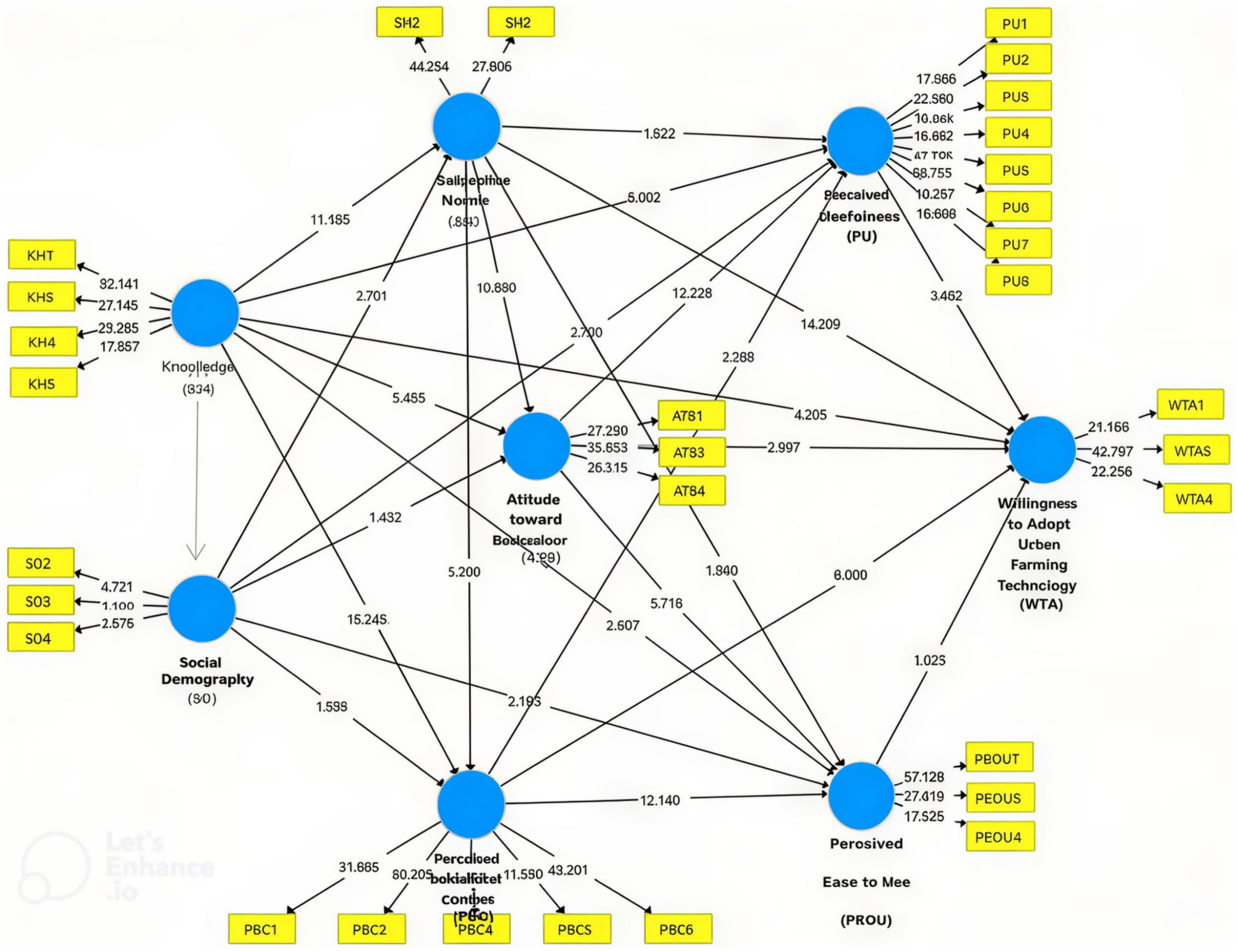
Bootstrapping results of the structural model.

Knowledge (KH) significantly influenced Subjective Norms (SN) and Attitude toward Behavior (ATB), both directly and indirectly via Perceived Ease of Use (PEOU) (*β* = 0.029, *p* = 0.000), but not through Perceived Usefulness (PU) (*p* = 0.641), as shown in Figure 7, and also directly shaped SN and ATB (*β* = 0.038, *p* = 0.006). SN affected Perceived Behavioral Control (PBC) and PEOU (*β* = 0.027, *p* = 0.000). At the same time, Social Demography (SD) indirectly influenced Willingness to Adopt Urban Farming (WTA) through PEOU (*β* = 0.024, *p* = 0.034), further shaping ATB, PBC, PEOU, and WTA with minimal effect on PU. Overall, KH, SN, and PBC played central roles, highlighting the psychological and social pathways driving WTA.

### 4.4 The model interpretation

#### 4.4.1 The direct influence of TAM/TPB variables on urban community acceptance regarding the willingness to adopt urban farming (UF)

The Analysis revealed that multiple factors influenced UF adoption in Makassar. Limited or incomplete knowledge reduced willingness to adopt, highlighting the need for clearer training and guidance. Social influence played a significant role, as encouragement from family or community members enhanced participation. Perceived behavioral control also mattered; individuals with higher self-efficacy were more inclined to adopt when tools and support were accessible. Positive attitudes strengthened adoption intentions, and technologies perceived as easy to use promoted wider community engagement.

#### 4.4.2 The direct influence of TAM/TPB variables on urban community acceptance regarding the willingness to adopt urban farming (UF)

Knowledge (KH) played a central role in shaping key constructs of the Technology Acceptance Model (TAM) and the Theory of Planned Behavior (TPB), which in turn influenced urban communities’ willingness to adopt Urban Farming (UF) technologies. The findings are described as follows:

1. Perceived behavioral control (PBC)

Greater knowledge enhanced Perceived Behavioral Control (PBC), as individuals with access to training, seminars, or digital resources felt more capable of managing UF adoption. A solid knowledge base improved their preparedness to face challenges, thereby increasing their willingness to adopt UF technologies, consistent with previous studies indicating that education strengthens readiness for technology adoption.

1. Perception of ease of use (PEOU)

This study identified a significant relationship between Knowledge (KH), Perceived Ease of Use (PEOU), and Willingness to Adopt Urban Farming (WTA). The findings indicated that a deeper understanding of UF technology enhanced perceptions of ease of use, which in turn increased the likelihood of adoption. In other words, individuals with greater knowledge were more likely to perceive the technology as user-friendly, thereby strengthening their willingness to apply it in practice. These results highlight the importance of comprehensive education and effective training to support community adoption of UF technologies.

1. Subjective norms (SN)

Knowledge (KH) significantly influenced Willingness to Adopt Urban Farming (WTA) through Subjective Norms (SN). SN played a pivotal role in shaping attitudes and intentions toward adopting new technology. When individuals received social support and guidance aligned with prevailing norms, they were more likely to adopt UF technologies. In Makassar, promoting positive norms through community campaigns and leadership support proved effective in encouraging UF adoption.

1. Subjective norms (SN) and perceived behavioral control (PBC)

Knowledge (KH) significantly influenced the adoption of UF technologies in Makassar by shaping subjective norms and enhancing individuals’ perceived control over technology use. SN, guided by community expectations, facilitated the integration of UF into daily routines. Programs led by local leaders, such as experienced farmers or environmental activists, provided practical insights into the benefits of UF for improving yields and promoting sustainability.

Moreover, structured educational initiatives, including training and workshops, enhanced technical knowledge and confidence, enabling communities to address practical challenges such as plant care and water management, while also fostering broader social support for adoption. Collaboration among communities, government agencies, and the private sector further reinforced this supportive environment, ultimately advancing the adoption of UF technologies and strengthening urban livelihoods.

1. Subjective norms (SN) and attitude toward behavior (ATB)

This study demonstrated that Knowledge (KH) substantially shaped individuals’ attitudes toward adopting UF technology in Makassar. SN, reflecting societal expectations, mediated the relationship between Knowledge (KH) and attitudes toward adoption. Respondents with higher knowledge levels were more likely to align with supportive social norms, thereby enhancing perceptions of UF’s usability. Consequently, favorable attitudes toward UF increased the likelihood of adoption. These findings are consistent with the Theory of Planned Behavior (TPB), which emphasizes the role of social norms in shaping intentions, and align with previous evidence on the importance of SN in technology adoption.

1. Subjective norms (SN), attitude toward behavior (ATB), and perceived ease of use (PEOU)

Knowledge (KH) also influenced SN, which, in turn, shaped ATB and subsequently affected PEOU, thereby enhancing willingness to adopt UF technology. In Makassar, social groups and community leaders played a central role in forming attitudes toward new practices. Supportive norms, promoted by local leaders and influencers, enhanced perceptions of UF technology’s ease of use. Community campaigns, training workshops, and institutional support from government and NGOs, including resource provision, further reinforced these norms. Collectively, these efforts strengthened community attitudes and facilitated broader acceptance of UF technology.

1. Subjective norms (SN), perceived behavioral control (PBC), and perceived ease of use (PEOU)

The findings further indicated that Knowledge (KH) influenced PBC through SN, thereby affecting PEOU. Social and cultural dynamics in Makassar shaped perceptions of UF technology, with community support enhancing self-regulation and readiness to adopt. Local mentors demonstrated practical ways to integrate UF into daily routines. Collaboration among local governments, NGOs, and the private sector expanded access to UF technology, aligned with the Sustainable Development Goals, and addressed barriers such as limited technical knowledge and financial constraints. Training initiatives and awareness campaigns reinforced positive norms, creating a supportive environment for adoption.

#### 4.4.3 The direct influence of TAM/TPB variables on urban community acceptance regarding the willingness to adopt urban farming (UF)

The analysis revealed that socio-demographic factors (SD) significantly affected PEOU in Makassar, with age, education, and socioeconomic status influencing perceptions of UF technology adoption. Higher education promoted acceptance, whereas advanced age and limited economic resources constrained it. Therefore, strategies to enhance UF adoption should prioritize awareness and educational programs tailored to diverse demographic groups.

#### 4.4.4 The role of perceived ease of use (PEOU) in mediating the influence of perceived behavioral control (PBC) on the willingness of individuals to adopt urban farming (UF)

The results indicated that PBC significantly influenced WTA through PEOU, with strong statistical support. Respondents who felt greater control over their actions were more confident in using UF technology, thereby increasing their likelihood of adoption. In Makassar, community-driven initiatives and educational programs that enhance perceptions of control proved essential. These findings underscore the critical role of psychological and social factors in technology adoption, consistent with earlier research emphasizing their impact on attitudes and behaviors toward innovation.

#### 4.4.5 The role of TAM/TPB variables in mediating the influence of subjective norm (SN) variables on the community’s willingness to adopt UF

To further understand the mediating mechanisms underlying the influence of subjective norms (SN) on the willingness to adopt urban farming (UF), a more detailed analysis was conducted. The analysis revealed several important relationships:

1. Attitude toward behavior (ATB) and perceived ease of use (PEOU)

The analysis revealed that SN, representing societal expectations, significantly influenced both ATB and PEOU. Support from family, peers, and community members shaped positive perceptions of UF, enhancing both favorable attitudes and perceptions of usability. These perceptions, in turn, increased adoption. Therefore, fostering supportive social norms and highlighting the practical benefits of UF are effective strategies to promote adoption in Makassar.

1. Attitude toward behavior (ATB)

The analysis emphasized the significant role of subjective norms in shaping attitudes toward UF adoption. Subjective norms, encompassing expectations and support from family, peers, and the broader community, strongly influenced individual attitudes toward the technology. Exposure to favorable norms increased the likelihood of developing positive attitudes, which in turn enhanced adoption intentions. Moreover, subjective norms mediated the relationship between attitude toward behavior and willingness to adopt, indicating that social influences not only directly shaped attitudes but also indirectly motivated technology adoption.

Based on these findings, strategies to promote UF adoption in Makassar should prioritize strengthening supportive social norms. Community outreach, educational campaigns, and engagement with local leaders can reinforce norms that encourage adoption. By fostering such norms, the willingness of Makassar’s residents to integrate UF technologies into sustainable urban lifestyles is likely to increase.

1. Perceived behavioral control (PBC) and perceived ease of use (PEOU)

The analysis showed that subjective norms significantly mediated the relationship between perceived behavioral control (PBC) and perceived ease of use (PEOU) of UF technology. These norms, shaped by the expectations and support of family, peers, and the broader community, enhanced adoption. Positive subjective norms increased individuals’ confidence in using UF technology, thereby strengthening perceptions of usability. To promote UF adoption in Makassar, reinforcing supportive norms through social campaigns, public awareness initiatives, and leadership engagement is crucial. Such efforts are expected to foster greater community willingness to adopt UF as a sustainable response to urbanization challenges.

1. Perceived behavioral control (PBC)

Subjective norms significantly influenced individuals’ intentions to adopt UF technology through perceived behavioral control (PBC). Shaped by the expectations and attitudes of the surrounding social context, these norms enhanced perceptions of individuals’ ability to manage and apply the technology. Supportive reinforcement from social networks increased confidence, thereby promoting adoption. In Makassar, strong subjective norms reinforced by community programs and influential agricultural figures strengthened PBC and encouraged adoption. Further evidence suggested that community-based educational initiatives and successful UF demonstrations improved self-efficacy, ultimately fostering broader technology adoption.

## 5 Discussions

This research provides a comprehensive understanding of the factors influencing community participation in urban farming practices. The results indicate that perceived usefulness and perceived economic benefits significantly impact the community’s intention to adopt urban farming. These findings suggest that perceptions of practical advantages and economic gains are crucial in promoting community involvement.

Consistent with the Technology Acceptance Model (TAM), perceived usefulness plays a pivotal role in shaping individuals’ attitudes toward urban farming. The higher the community’s perception of urban farming’s usefulness, the more positive their attitudes, ultimately increasing their intention to participate. Furthermore, within the Theory of Planned Behavior (TPB) framework, variables such as attitude toward behavior, subjective norms, and perceived behavioral control also significantly influence behavioral intentions. This highlights that both internal cognitive factors and external social influences are essential to encouraging the adoption of urban farming.

Compared to previous literature, these findings align with studies emphasizing the importance of economic benefits and social support in fostering community participation in urban agriculture. Moreover, this study underscores the role of knowledge and sociodemographic characteristics in shaping adoption, aspects that have not been comprehensively addressed in some prior research. Enhancing literacy and public understanding of urban farming is thus a critical consideration for policy development. Overall, this study contributes by integrating TAM and TPB approaches within the context of urban farming in Indonesia, reinforcing the validity of both models while providing novel insights on economic, social, and knowledge factors that drive successful community-level implementation.

## 6 Policy implications for enhancing public willingness to adopt urban farming (UF)

**Figure 8:**
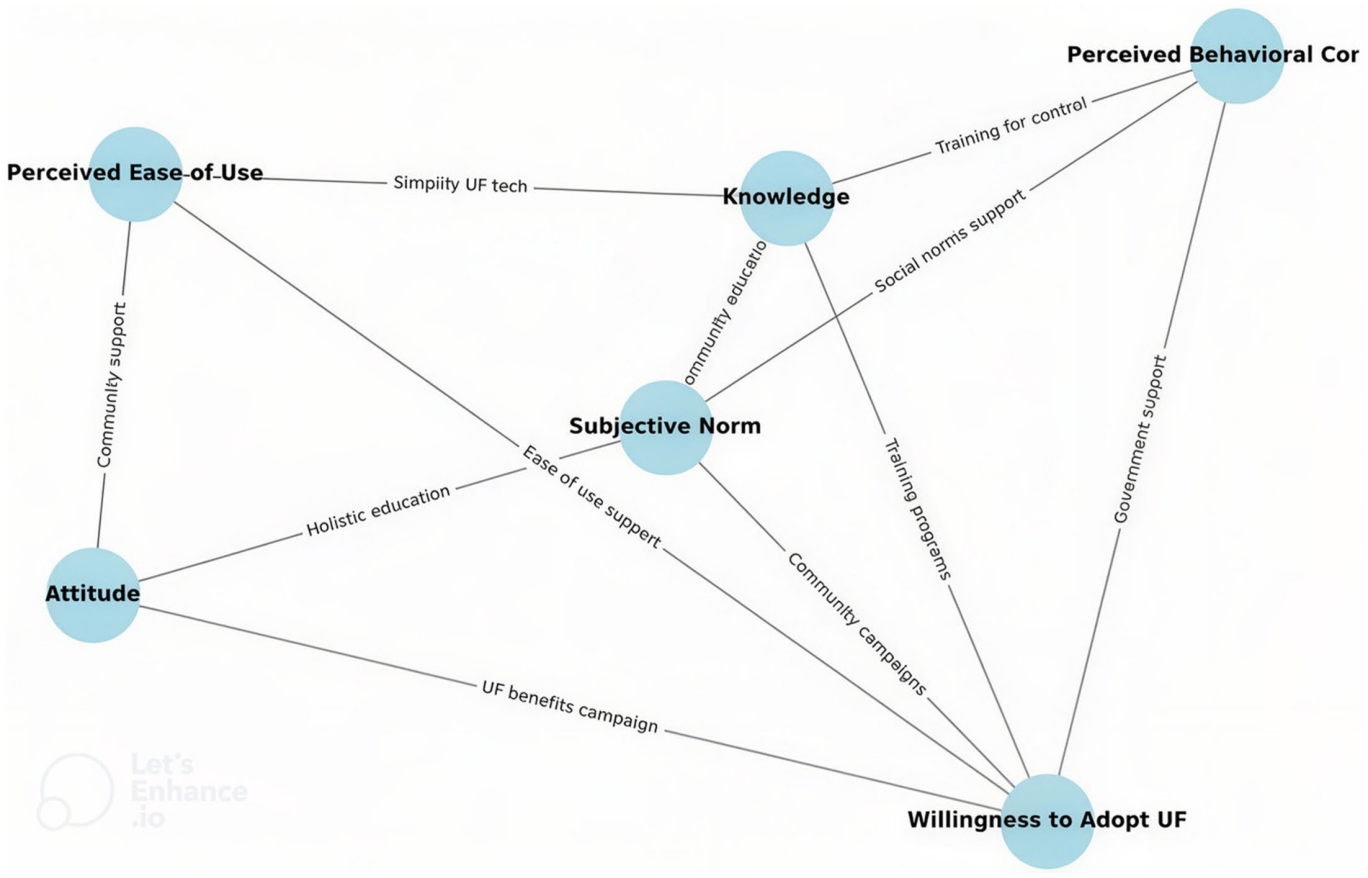
Policy framework for urban farming adoption.

This study emphasizes the importance of targeted policies to promote the adoption of urban farming (UF). As shown in Figure 8, training programs enhanced knowledge, which in turn influenced social norms, attitudes, and perceived control, ultimately increasing adoption. Community campaigns and educational initiatives further reinforced these drivers of participation. For effective policy design, government support should prioritize accessible and sustainable interventions, including subsidized training, public awareness campaigns, and financial incentives. Collectively, these measures are expected to foster stronger community engagement and broader acceptance of UF.

## 7 Conclusion

This study examined the direct and indirect effects of Knowledge and Social Demography on Perceived Ease of Use and Perceived Usefulness within the Technology Acceptance Model (TAM), alongside the effects of Attitude, Subjective Norms, and Perceived Behavioral Control from the Theory of Planned Behavior (TPB). Using SEM-PLS, the findings identified several factors shaping the willingness to adopt urban farming (UF) technology in Makassar.

First, knowledge positively influenced perceptions of ease of use, although excessive technical detail may raise concerns that discourage adoption, highlighting the need for balanced educational approaches. Second, subjective norms within the social context strongly shaped attitudes and intentions, emphasizing the importance of family, peer, and community support. Third, perceptions of user-friendliness were crucial, with greater accessibility and training increasing the likelihood of adoption. Finally, the integrated use of TAM and TPB proved effective in predicting UF adoption, particularly in contexts with small sample sizes or non-normal data distributions.

These results suggest that promoting UF adoption in Makassar requires a comprehensive strategy that combines balanced education, reinforcement of positive social norms, and the provision of supportive infrastructure. Beyond Makassar, the findings contribute to a broader understanding of technology adoption in urban agriculture and provide a foundation for designing targeted interventions in other urban contexts. Future research could extend this analysis to different cities, comparing demographic, social, and technological dynamics to refine strategies for fostering sustainable UF adoption.

## Data Availability

The raw data supporting the conclusions of this article will be made available by the authors, without undue reservation.
